# Local coexistence of VO_2_ phases revealed by deep data analysis

**DOI:** 10.1038/srep29216

**Published:** 2016-07-07

**Authors:** Evgheni Strelcov, Anton Ievlev, Alex Belianinov, Alexander Tselev, Andrei Kolmakov, Sergei V. Kalinin

**Affiliations:** 1 Institute for Functional Imaging of Materials and Center for Nanophase Materials Sciences, Oak Ridge National Laboratory, Oak Ridge, Tennessee 37831, United States; 2 Center for Nanoscale Science and Technology, National Institute of Standards and Technology, Gaithersburg, MD 20899, USA; 3 Maryland Nanocenter, University of Maryland, College Park, MD 20742, USA.

## Abstract

We report a synergistic approach of micro-Raman spectroscopic mapping and deep data analysis to study the distribution of crystallographic phases and ferroelastic domains in a defected Al-doped VO_2_ microcrystal. Bayesian linear unmixing revealed an uneven distribution of the T phase, which is stabilized by the surface defects and uneven local doping that went undetectable by other classical analysis techniques such as PCA and SIMPLISMA. This work demonstrates the impact of information recovery via statistical analysis and full mapping in spectroscopic studies of vanadium dioxide systems, which is commonly substituted by averaging or single point-probing approaches, both of which suffer from information misinterpretation due to low resolving power.

Vanadium dioxide is a classic example of a strongly-correlated electron system exhibiting a near room temperature uniquely-sharp metal-insulator transition (MIT) that drastically changes the electronic and optical properties of this material. The MIT defines the wide range of existing and potential applications of VO_2_ in microbolometers^1^,^2^, smart windows^3^, thermochromic devices^4^, nanoactuators^5^,^6^,^7^, ultra-fast optical switches^8^, sensors^9^,^10^, field-effect transistors^11^,^12^,^13^,^14^,^15^ etc. Control over MIT is gained via temperature, elastic stress, electric field and doping. The latter method can be used for tuning of the MIT temperature and for stabilization of a range of crystallographic phases: from the monoclinic M1 and tetragonal R, stable in pure VO_2_ at ambient pressure, to metastable monoclinic M2 and triclinic T. The Giznburg-Landau phase transition theory describes the lower-symmetry VO_2_ phases as different structures which can be realized as a result of rutile lattice instabilities at the two non-equivalent R-points of the Brillouin zone^16^. This phase interdependence substantiates investigation of the low-symmetry phases that may shed light on the controversial question of the origin of MIT in vanadium dioxide^17^,^18^,^19^.

Confocal micro-Raman spectroscopy is a versatile and non-invasive tool that has been extensively used for *in situ* identification of local specific crystallographic phases of VO_2_ and their transformations as a function of temperature or mechanical stress. However, its use in VO_2_ surface mapping has been limited. Only a few groups have utilized micro-Raman mapping of VO_2_ crystal/film surfaces^16^, or samples containing VO_2_/V_2_O_5_ nanowires^20^ to fully unravel the spatial distribution of specific phases. Commonly, simpler approaches such as spectral averaging over a large area or point-probing the sample^21^,^22^ (e.g. measuring spectra at selected locations along a VO_2_ nanobeam length) have been used. Needless to say, micro-Raman spectroscopy offers much richer spectral content that can be used for solving the important question of phase separation (see ref. 23 as an example). However, in many complex multi-phase systems, local conditions may stabilize phases beyond those that exist and have been characterized in the bulk. Hence there is the need for the blind signal unmixing methods that can help to determine the spatial distributions and to deconvolute the spectra of unknown phases. Here we employ a combination of chemometric blind unmixing tools and micro-Raman spectroscopy to investigate the partition of phases in a VO_2_ microbelt under uneven distribution of stress and dopant concentration.

## Results and Discussion

Al-doped VO_2_ nanostructures were grown on Si/SiO_2_ substrates according to the protocol described earlier^24^. A low dopant concentration was used to bring the system into the broad domain of M1/T stability in the temperature-composition phase diagram (see Fig. 1 in ref. 24), where an interesting interplay of these two phases can be observed at room temperature. The growth process involves dissolution of alumina in molten V_2_O_5_ microdroplets condensed on the Si/SiO_2_ substrate^25^, with the subsequent crystallization of Al-doped VO_2_ nanostructures from them. The grown nanostructures (nanowires, nanorods, nano and microbelts) are typically single crystals of highest quality, as evidenced by x-ray studies and the sharpness of MIT^24^. However, occasionally, the local growth conditions (precursor influx, Al concentration, temperature gradient, etc.) favor precipitation of secondary nanocrystal layer on the surface of the previously formed VO_2_ structures. These newly formed crystals have a rough surface and present a scientific opportunity to model non-ideal VO_2_ crystals with uneven distribution of stress and dopant concentration. It also has a practical importance in understanding of role of the crystal defects and strain on performance of VO_2_-based devices.

**Figure 1:**
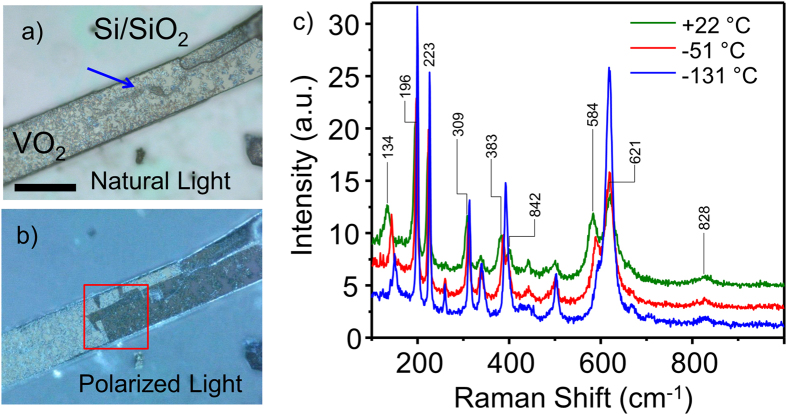
(**a**) Optical micrograph of a VO_2_ microbelt grown on a Si/SiO_2_ substrate as recorded in natural light; the blue arrow indicates a surface defect; the scale bar is 50 μm, (**b**) Image of the same area under crossed Nicols illumination showing ferroelastic domains; the red square highlights the region of interest, (**c**) Temperature dependence of averaged Raman spectra recorded from a single-crystal free-standing nanobelt grown on the same substrate as the one shown in panels (**a,b**) a clear transition from T phase to M1 phase is observed upon cooling. Peaks are indexed only for the spectra recorded at room temperature, as only they are used later for comparison. Spectra are displayed with a y-offset for clarity.

**Figure 2:**
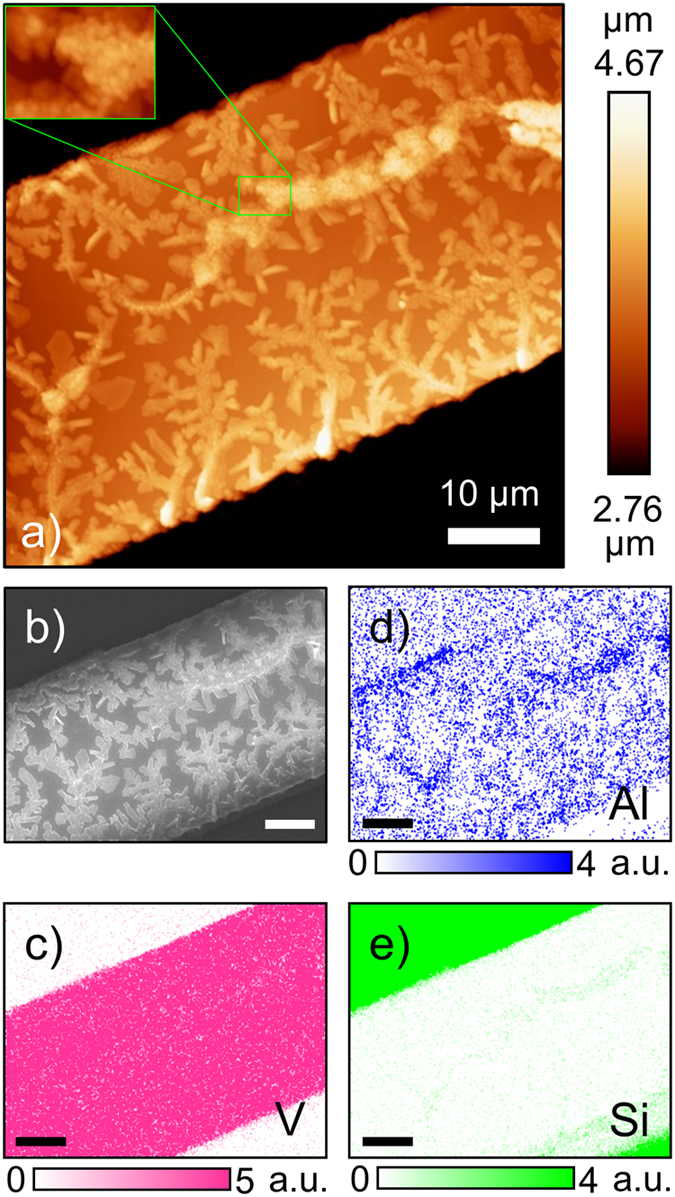
The maps of the region of interest: (**a**) tapping mode AFM topographic image, (**b**) SEM micrograph, and the corresponding (**c**) vanadium, (**d**) aluminum, and (**e**) silicon EDX maps highlighting concentration of Al and Si in the deposit. Scale bar in (**b**–**e**) is 10 μm.

An optical image of such a microbelt embedded into the growth substrate is shown in Fig. 1a. It is hundreds of microns long, 43 μm wide and ca. 3.2 μm thick, making it a convenient object for micro-Raman mapping. After the growth, the microbeam remains under a substrate-induced axial tensile stress. To minimize the strain energy, a system of ferroelastic domains is usually formed which can be easily accessed with polarized light microscopy (Fig. 1b). The surface of the Al-doped VO_2_ microbelt is covered with precipitated crystals that form dendritic efflorescence-shaped patterns and a large bowed defect running along the middle of the top crystal facet (blue arrow, Fig. 1a). The defect does not seem to correlate with the ferroelastic domain structure appearing close to the straight domain wall. A closer examination of the deposit with Atomic Force Microscopy (AFM) shows that it is ca. 250 nm tall, reaching 600 nm to 800 nm in the bowed defect (Fig. 2a). The defect itself is comprised of conglomerates of clumped nanocrystals 100 nm to 400 nm in diameter (Fig. 2a, inset). In order to clarify the chemical composition of the deposit, we performed Energy Dispersed X-ray (EDX) analysis of the micro-belt, which revealed a high Al and Si content in the precipitate. The chemical maps of V, Al and Si are displayed in Fig. 2(c–e). These maps illustrate that while vanadium is only found in the microbelt, aluminum is present both in the microbelt and in the substrate, concentrating in the deposit (especially in the bowed defect); in addition, surprisingly, small quantities of silicon were detected in the bowed defect as well. Taking into account that previous study^24^ of high-quality Al-doped VO_2_ nanobelts excluded the possibility of Si doping of VO_2_, we attribute the Si signal exclusively to the deposit. The crystalline form of the deposit is also very different from that of alumina that re-crystalizes from molten vanadia^24^, which makes it unlikely to be comprised of Al_2_O_3_. Furthermore, the deposit is clearly more electrically insulating than the underlying VO_2_ microbelt, as evidenced by the surface charging during SEM imaging (Fig. 2b). Thus, we conclude that the precipitated nanocrystals might be comprised of heavily-doped VO_2_, aluminum vanadates/vanadites (AlVO_4_, AlVO_3_, etc.) with an admixture of alumosilicates. It is worth noting that in the regions underneath the deposit the microbelt could be more heavily doped with Al than on the average, and that the deposit can exert unevenly-distributed local stress on the microbelt surface (see Figure S3 and discussion in Supplemental Information). Both of these factors are known to influence the VO_2_ crystallographic phase stability at a given temperature. Therefore, spatial mapping of the phase distribution would be particularly helpful in this system.

Micro-Raman measurements were performed in the region of interest of the microbelt, (Fig. 1b, red square) containing the bowed defect using a joint AFM/spectroscopic platform. The diode-pumped solid-state laser (λ = 532 nm) power was minimized to about 20 μW to avoid local overheating and related MIT/oxidation. The laser polarization plane (linear polarization) was set perpendicular to the microbelt length. A 60 μm × 60 μm region was mapped out at a spatial resolution of 100 × 100 pixels, producing a 100 × 100 × 1650 hyperspectral datacube. Visualization of n-dimensional datasets is possible by employing statistical tools, such as principal component analysis (PCA), independent component analysis, k-means clustering, etc.^26^ However, these and other similar methods yield results that, although statistically correct, are hard to interpret physically. Figure S1 of Supplemental Materials shows PCA decomposition of the Raman dataset into the loading maps and corresponding eigenvectors, revealing the difference between the Raman signals of the oppositely-oriented ferroelastic domains and existence of a separate region (Figure S1, 4) with a unique Raman signature, absent in the optical, AFM or SEM micrographs. However, the spectrum corresponding to this region cannot be easily identified, as PCA eigenvectors are not physically constrained and cannot be directly compared to the Raman spectra of pure phases. Another common chemometric tool used for deconvoluting Raman datacubes is the spectral unmixing via the SIMPLISMA algorithm^27^,^28^. SIMPLISMA assumes existence of a chemical variable (e.g. spectral intensity at a certain wavenumber) in the dataset that experiences a major influence from only one of the components (materials) in the mixture. Thus, by identifying such a pure variable, the spectral mixture can be resolved into individual spectra of components (for more details, see SI). As seen in Figure S2, SIMPLISMA easily unmixes the Raman datacube into the VO_2_ and silicon substrate signals (2 components), but further separation of ferroelastic domain spectra (3 components) and the third phase spectra (4 components) fails. Therefore, here we employed the recently-developed Bayesian Linear Unmixing (BLU) algorithm^29^,^30^ to fully unmix the intricate spectroscopic behavior of the microbelt.

**Figure 3:**
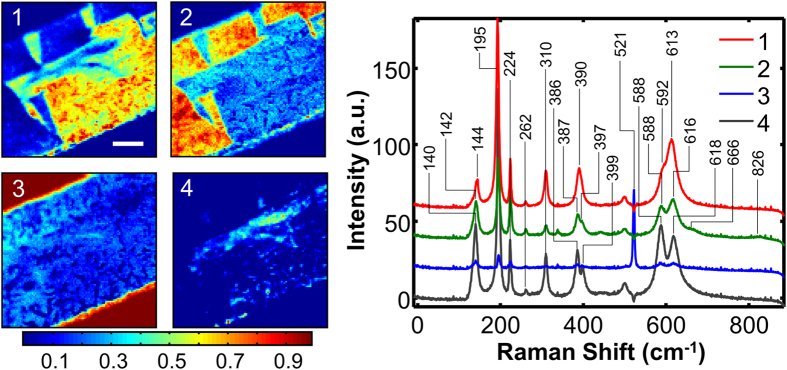
BLU of Raman spectroscopic data into 4 components: 2 ferroelastic domains (shown in panels 1 and 2), Si substrate (panel 3) and T-phase regions (panel 4). Abundance maps (intensity as a fraction of unity) and corresponding endmember spectra are shown. The scale bar is 10 μm. Spectra are displayed with a y-offset for clarity.

The BLU method considers data *Y* as a linear combination of position-independent endmembers, *M*, with respective relative abundances, *A*, corrupted by additive Gaussian noise *N*: *Y* = *MA* + *N*. This method incorporates several built-in constraints that allow physical interpretation of results: the non-negativity (*M*_*i*_ ≥ 0, *A*_*i*_ ≥ 0), full additivity and sum-to-one (∑*A*_*i*_ = 1) constraints for both the endmembers and the abundance coefficients. To solve the blind unmixing problem, the BLU algorithm estimates the initial projection of endmembers in a dimensionality reduced subspace (PCA) via N-FINDR – a geometrical method that searches for a simplex of maximum volume that can be inscribed within the hyperspectral data set using a simple non-linear inversion. The endmember abundance priors as well as noise variance priors are then chosen by a multivariate Gaussian distribution, where the posterior distribution is calculated based on endmember independence using Markov Chain Monte Carlo that generates asymptotically distributed samples probed by Gibbs sampling strategy. Due to non-negativity of the resulting endmembers *M* and normalization of abundances, the spectrum at each location is decomposed into a linear combination of spectra of individual components in corresponding proportions. The number of spectral components must be provided by the researcher and can be estimated using PCA or by under- and oversampling. A detailed description and testing of BLU against PCA, k-means and other statistical methods can be found elsewhere^31^,^32^. The PCA maps (Figure S1) suggest that there are 4 to 5 components, corresponding to the Si substrate, 2 types of ferroelastic domains, and 1 to 2 other phase regions. Bayesian unmixing of the datacube into 4 components is shown in Fig. 3. As can be seen, a proper separation of the 2 ferroelastic domains (Fig. 3: 1 to 2), substrate (Fig. 3: 3) and a third phase (Fig. 3: 4) is achieved. The corresponding endmembers (unmixed spectra of pure components) can be directly compared to the spectra of individual materials and are easily identifiable. Endmembers 1 and 2 correspond to the Raman spectra of the M1 phase ferroelastic domains, as identified by their shapes and spatial distribution. They mostly differ by the relative intensity of the V-motion modes^33^ at 195 cm^−1^ and 224 cm^−1^, the former decreasing in intensity, the latter staying constant. Both of these modes have A_g_ symmetry and a non-circular dependence of intensity on the polarization angle^20^. Endmember 3 shows one strong peak at 521 cm^−1^ originating from the silicon substrate. The most intriguing fourth component, localized in the vicinity of the bowed defect, can now be tentatively identified by comparing its spectrum to the spectra of the known VO_2_ crystallographic phases.

**Figure 4:**
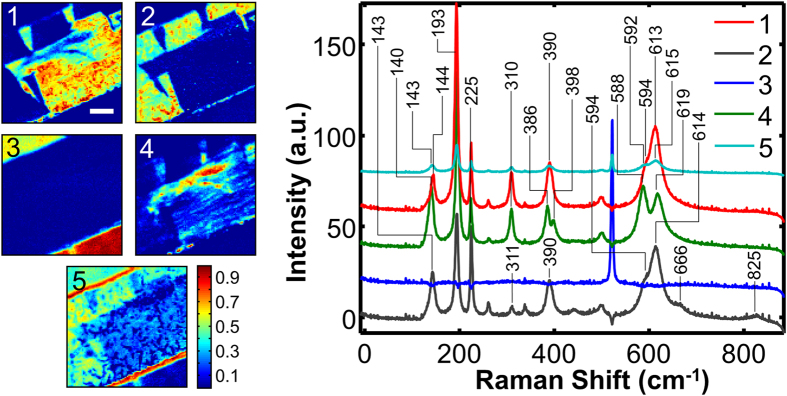
BLU of Raman spectroscopic data into 5 components: 2 ferroelastic domains, Si substrate, T-phase and interfacial regions. Abundance maps (intensity as a fraction of unity) and corresponding endmember spectra are shown. The scale bar is 10 μm. Spectra are displayed with a y-offset for clarity.

Figure 1b presents a temperature dependence of the averaged Raman spectra recorded from a free-standing single-crystal nanobelt released and collected from the same substrate as the studied microbelt and having similar doping level (2‰ to 3‰). At room temperature the nanobelt’s phase can be clearly identified as T, and a gradual transition to the M1 phase is observed upon cooling to −131 °C. The spectral signatures distinguishing these two phases at room temperature are: a) appearance of a T-phase peak at 584 cm^−1^, b) bifurcation of the M1 peak at 390 cm^−1^ into two T-peaks at 383 cm^−1^ and 401 cm^−1^, c) appearance of a small T-peak at 443 cm^−1^, and d) a bathochromic shift of an M1-peak from 143 cm^−1^ to 126 cm^−1^. Hence, the fourth Bayesian component can be tentatively identified as the VO_2_ T-phase. Recalling that the M1-T transition is gradual (unlike other phase transitions in VO_2_ that are sharp)^24^, characterized by a continuous distortion of the monoclinic lattice to the triclinic, we expect to see some intermixture of signatures. Indeed, the lower-frequency peak of component 4 appears not at 126 cm^−1^, but at 140 cm^−1^, and the peaks of component 2 around 600 cm^−1^ and 400 cm^−1^ appear to be slightly bifurcated, which is similar to the case of the nanobelt spectrum recorded at -51 °C (Fig. 1b), when transition to the M1 phase was not yet completed. As discussed before, this presents an additional challenge for the spectral unmixing algorithms, as the pure component spectrum can be very similar to a linear combination of the spectra of other components. Several tokens in Fig. 3 indicate that the unmixing may not be optimal: the Si signal (component 3) has non-zero intensity on the microbelt itself, and the corresponding endmember has several small peaks originating from VO_2_; component 3 map appears to have contributions from both types of the ferroelastic domains; component 2 has pronounced peaks at 588 cm^−1^ and 397 cm^−1^. In addition, the average abundance of the fourth component in the region of its highest intensity is only about 50%. Thus, in order to ensure the effectiveness of the BLU, we performed deconvolution of the datacube into five components. As can be seen (Fig. 4), this allowed complete separation of the ferroelastic domain signals and a better highlighting of component 4 by increasing its average abundance to ca. 80%. The ferroelastic domain signal of component 2 lost the 397 cm^−1^ mode and its 594 cm^−1^ peak decreased in intensity to the level typically observed in the M1 phase. The component 3 spectrum became completely void of VO_2_ peaks and has zero intensity on the microbelt. The mixed Si-VO_2_ signal was adjudicated into a separate component 5, whose intensity is around 90% on the microbelt edges. Further increase in the number of components is not feasible, as it brings about a physically-meaningless splitting of the existing components.

Interestingly, comparison of the component 4 maps to the AFM and SEM images indicates that although the T-phase region must be pinned by the bowed defect, it is in no way identical in shape with the later. The bowed defect concaves toward the top of the image, whereas the T-region convexes in the same direction (Fig. 4,4). Moreover, the T-region features a secondary bow that curves in the opposite direction (to the bottom of the image), which has no direct correspondence in the topographic images. We speculate that the T phase stabilization in this second bow is not driven by the local stress exerted by the precipitate on the microbelt surface, but by the local uneven Al doping inside the microbelt.

## Conclusions

To summarize, we have studied distribution of crystallographic and ferroelastic phases in a defected Al-doped VO_2_ microcrystal employing a synergistic conjunction of micro-Raman and Bayesian linear unmixing algorithm. The deep data analysis allowed discriminating an uneven distribution of the T phase, partly correlated with the surface defects. Stabilization of the T phase is likely due to the defect-induced stress and local uneven doping. This work demonstrates the importance of full mapping in hyperspectral micro-Raman studies of vanadium dioxide systems, which is commonly substituted with averaging or single point-probing approaches that suffer from low resolving power.

From a more general point of view, full data acquisition is required in order to increase the scientific output of the existing materials characterization techniques. However, that brings about the challenge of data analysis, visualization and interpretation. The big data and deep data statistical approaches can solve this problem by unraveling the information on the functional properties present in the raw data without losing unsought-for signatures of serendipitous phenomena. The deep data methods also allow for deconvolution of concurrent processes and for ascribing physical models to the statistically-relevant behaviors. The continuing progress in information and computer technologies suggests that the usage of these methods and their applicability to the areas of physics, chemistry and materials science will significantly expand in the nearest future, boosting the progress in materials design.

## Additional Information

**How to cite this article**: Strelcov, E. *et al*. Local coexistence of VO_2_ phases revealed by deep data analysis. *Sci. Rep.* **6**, 29216; doi: 10.1038/srep29216 (2016).

## Supplementary Material

Supplementary Information
