# Supplementary material for: Local coexistence of VO2 phases revealed by deep data analysis
Source: Sci Rep. 2016 Jul 7;6:29216. doi: 10.1038/srep29216 (PMC4935893; doi:10.1038/srep29216)
Supplement: Supplementary Information [file srep29216-s1.pdf]

Local coexistence of VO_2 phases revealed by deep data analysis

Evgheni Strelcov,[1,2,3,†] Anton Ievlev,[1] Alex Belianinov,[1] Alexander Tselev,[1] Andrei Kolmakov,[2] and Sergei V. Kalinin[1]

1. *Institute for Functional Imaging of Materials and Center for Nanophase Materials Sciences, Oak Ridge National Laboratory, Oak Ridge, Tennessee 37831, United States.*
2. *Center for Nanoscale Science and Technology, National Institute of Standards and Technology, Gaithersburg, MD 20899*
3. *Maryland Nanocenter, University of Maryland, College Park, MD 20742.*

Supporting Information

Principle component analysis

Principle component analysis (PCA) is a linear transformation of a 3D data set of $N \times M$ pixels formed by spectra containing P points into orthogonal components ranked by the degree of variability in them (in our case, $N = M = 100$, $P = 1650$). The first eigenvector contains the most variability within the spectral-image data; the second contains the most variable response after the subtraction of variance of the first from the first one, and so on. As a result, the several first loading maps contain bulk of information within the dataset, while the remaining sets are dominated by outliers. The number of significant elements can be chosen based on the overall shape of the eigenvector dependence or from correlation analysis of loading maps, paired to each of the eigenvectors. It is important to note that full PCA decomposition is the linear transformation of the data and hence no information loss occurs in the data set. That said, truncation of PCA expansion at significant components is irreversible process, equivalent to the data smoothing.

Figure S1 shows the first 9 components of PCA-decomposition of the Raman datacube. The first loading map highlights the microbelt, the second – Si substrate, the third – one type of the ferroelastic domains, and the fourth one – the T-phase region. Maps 5 and 6 have low intensity and single out some surface defects and microbelt edges. The higher-order loading maps are clearly noise-dominated. Only the first two eigenvectors are identifiable: the first one resembling the negative of the average M1 spectrum, and the second clearly being the Si spectrum. Eigenvectors 3 to 6 have high signal-to-noise ratio, but possess negative spectral regions and cannot

† Corresponding author: Evgheni.strelcov@nist.gov.

be directly compared to the standard Raman spectra. Consequent eigenvectors mostly contain noise and present no interest. Thus, although the PCA analysis is able to unmask the presence of the new phase region (loading map 4), it is useless in identification of the phase and quantifying its abundance.

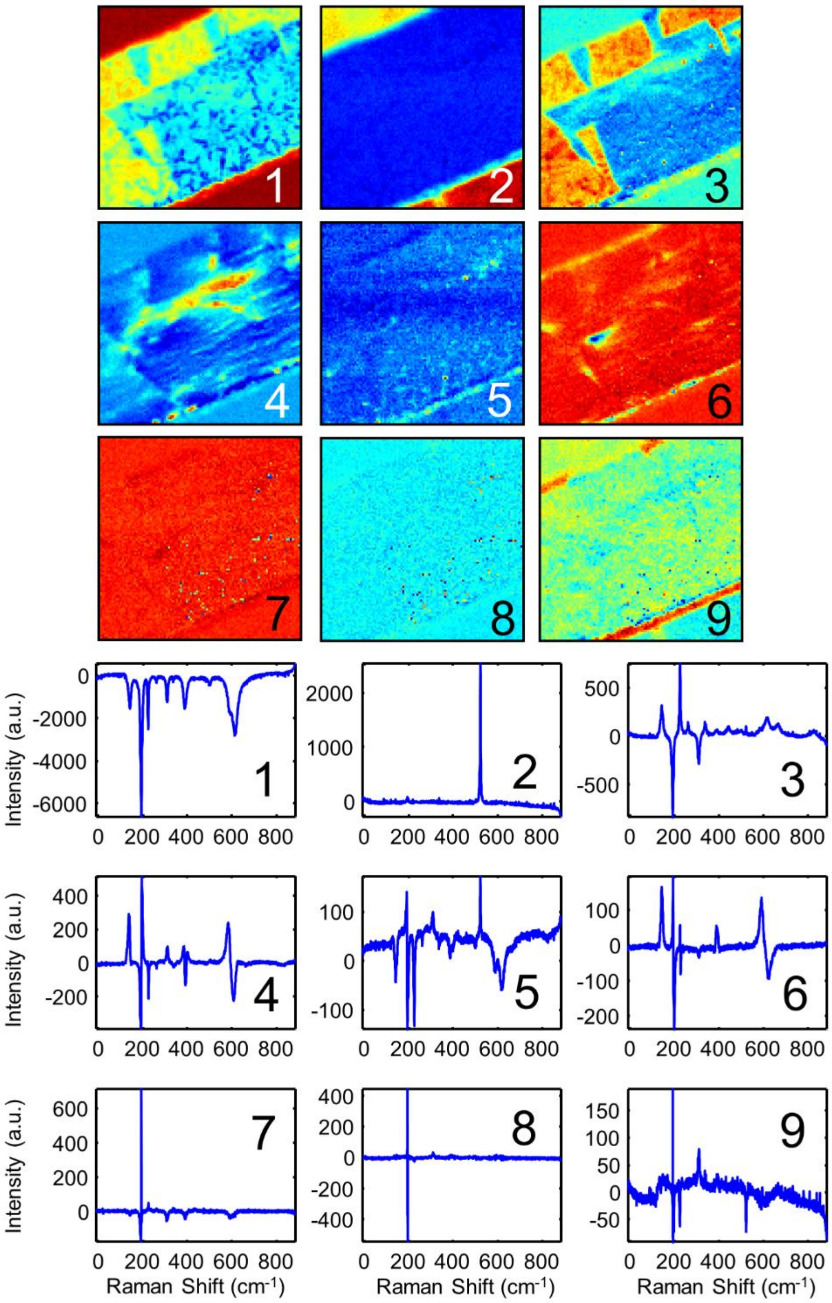

Figure S1. PCA of Raman spectroscopic data: the first 9 loading maps and corresponding eigenvectors are shown.

SIMPLISMA unmixing

The SIMPLISMA algorithm[1] assumes existence of a chemical variable (e.g. spectral intensity at a certain wavenumber) in the dataset that experiences a major influence from only one of the components (materials) in the mixture. The pure variables are sequentially identified according to the maximum of the function:

$p_{i,j} = \left[\frac{\sigma_i}{\mu_i+\alpha}\right]\omega_{i,j}$, , where $p_{i,j}$ is the purity value of the ith variable (in our case $1 \geq i \leq 1650$), from which the jth pure variable will be selected, σ is the standard deviation, μ is the mean of a chemical variable (over all measurement points), α is the offset, and $\omega_{i,j}$ is a determinant-based weight factor that corrects for the previously selected pure variables. The total number of selected pure variables L ($2 \geq j \leq L$) must be supplied by the researcher and, just like in case of BLU, can be determined from PCA or by over and undersampling. Following the selection, a purity spectrum is obtained in the form the p-vector, and the original datset Y ($N{\cdot}M{\times}P$) can be deconvoluted into abundance maps A ($N{\cdot}M$) and spectra matrix S, comprised of L-1 p-vectors: $Y = A{\cdot}S$. At the last stage, both spectra and abundance maps are normalized. Here, for SIMPLISMA umixing we adapted a Matlab code written by Kateryna Artyushkova and available for download as a part of a Multivariate Image Analysis of Multispectral Images GUI at http://www.unm.edu/~kartyush/matlab.xhtml.

Figure S2 presents unmixing of the Raman datacube into 2, 3 and 4 components using SIMPLISMA. Deconvolution of the VO_2 and Si signals is clear in the loading maps 1 and 2. The corresponding endmembers look like the Raman spectra of M1 phase and Si with the other component subtracted and can be easily identified. Splitting the dataset into 3 components allows highlighting the spatial distribution of the two ferrolastic phases (1’ and 3’, Figure S2) and Si substrate (2’, Figure S2). However, the ferroelastic domain spectra are unrealistically exaggerated, with the VO_2 peaks at 195 cm^{-1} and 224 cm^{-1} completely subtracted from the endmembers 3’ and 1’, correspondingly. The real Raman spectra collected from oppositely-oriented domains only feature decreases in intensities of these peaks relative to each other, and never their complete absence. Note, also, that the abundance of component 1’ doesn’t exceed 15 % anywhere in the sample, which is indicative of oversampling for this method. Still, the overall unmixing quality is sufficient for spectra identification. Finally, in order to extract the third phase region (i.e. the T phase), unmixing into 4 components was performed. It is known that SIMPLISMA is very sensitive to oversampling, often producing negative abundances and/or spectra if a high L value is chosen[2]. Indeed, at L = 4, one of the unmixed components was assigned negative abundances and spectral values, for which reason we introduced a non-negativity constraint to SIMPLISMA. The results are shown in Figure S2. Abundance map 4” correctly captured the spatial distribution of the T-phase, but its endmember can hardly be identified as the Raman spectrum of T-VO_2. In addition, the maximal abundance of component 1” is only 2%, and endmember 1” is quite exaggerated. We note that the partial failure of the SIMPLISMA algorithm, which was shown to be very effective when applied to other systems, presumably stems from the fact that the spectra of VO_2 phases (components) differ by position and relative intensity of several peaks, whereas SIMPLISMA have repeatedly chosen only the 195 cm^{-1} and 224 cm^{-1} peaks as pure variables. These are good for separating the two M1-phase ferroelastic domain regions, but not the T-phase that differs by the bifurcating peaks around 400 cm^{-1} and 600 cm^{-1}, which were never selected as pure variables. BLU, on the other hand, evaluates not individual variables (peaks), but the whole endmember in one single step, taking into account its overall shape. Hence, it proved to be more effective when applied to the present system.

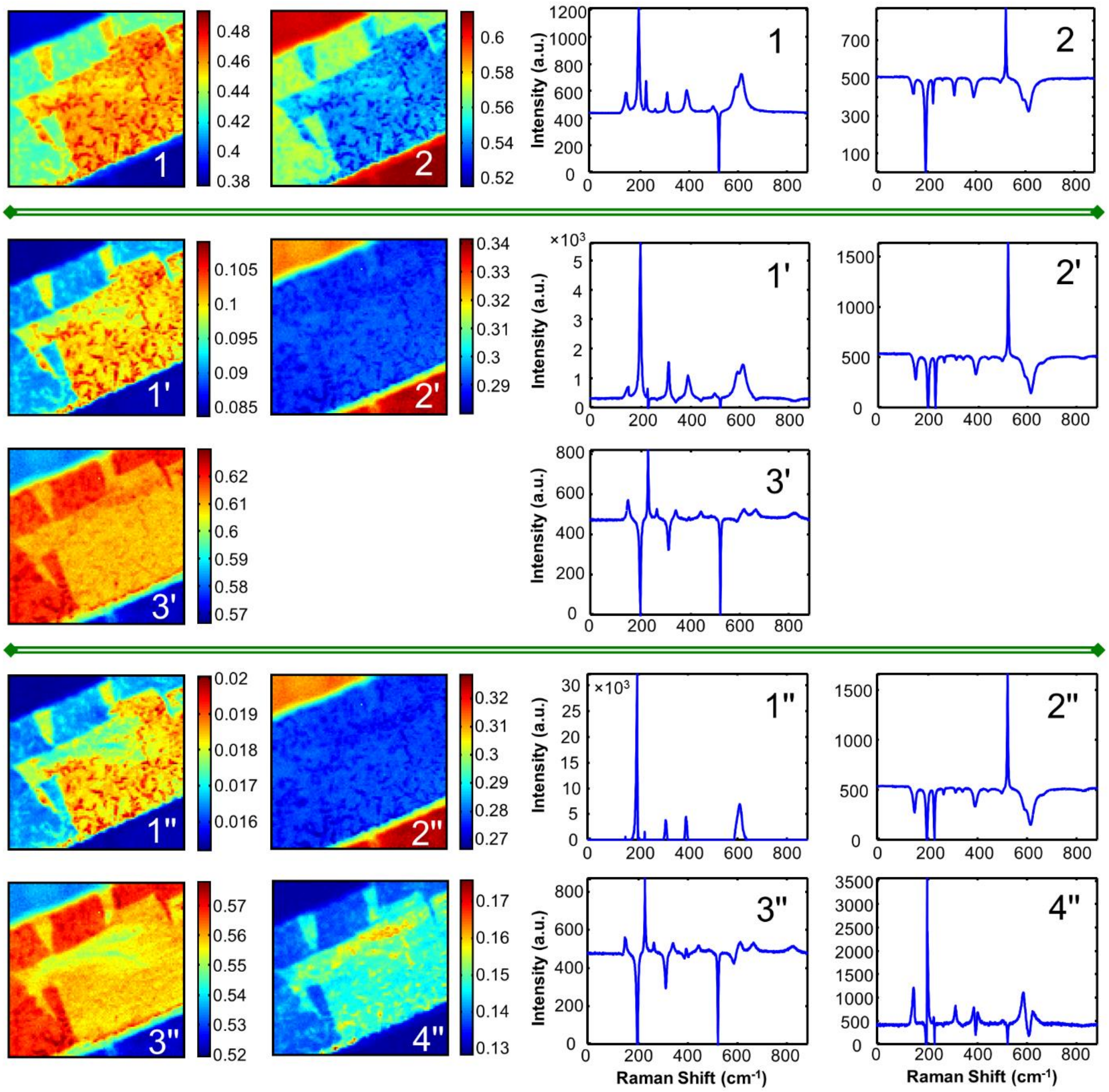

Figure S2. SIMPLISMA umixing of Raman spectroscopic data into 2, 3 and 4 components. Loading maps (intensity as a fraction of unity) and corresponding endmembers are shown.

Strain estimates

The collected spectral Raman data can be used for strain mapping. Local strain is proportional to frequency shifts in several Raman modes of VO_2. However, conversion of frequency shifts into strain values is significantly complicated by the compleplexity of the investigated system, where a non-trivial interplay between the biaxial strains originating in substrate clamping, nanocrystals and doping exists. Still, an approximate estimate of the existing strain can be done by calibrating shifts agains known literature data. Wu's group performed[3] a direct correlation of the Raman shifts and uniaxial strain applied to VO_2 microbeams. Figure S3 presents relative strain maps of the microbelt used in our study. Frequency shifts of vanadium mode (V1) at 193 cm^{-1} and oxygen mode at 611 cm^{-1} were extracted by Lorentzian fit of the raw spectral data and then converted to uniaxial strain using Figure 2d and 2e of Reference[3] as calibration curves. Unstrained values of the peak positions were taken as 192.9 cm^{-1} and 610.7 cm^{-1}, respectively. As can be seen, Figure S3 supports the main text assumption on the spatial distribution of strain and its concentration in the vicinity of the bowed defect, where it stabilizes the T-

phase. Note, that since the V1 mode frequency is less affected by stress than the O mode, Fig. S3a map has lower spatial resolution than S3b, even though these maps are plotted from the same datacube with a uniform spectral resolution.

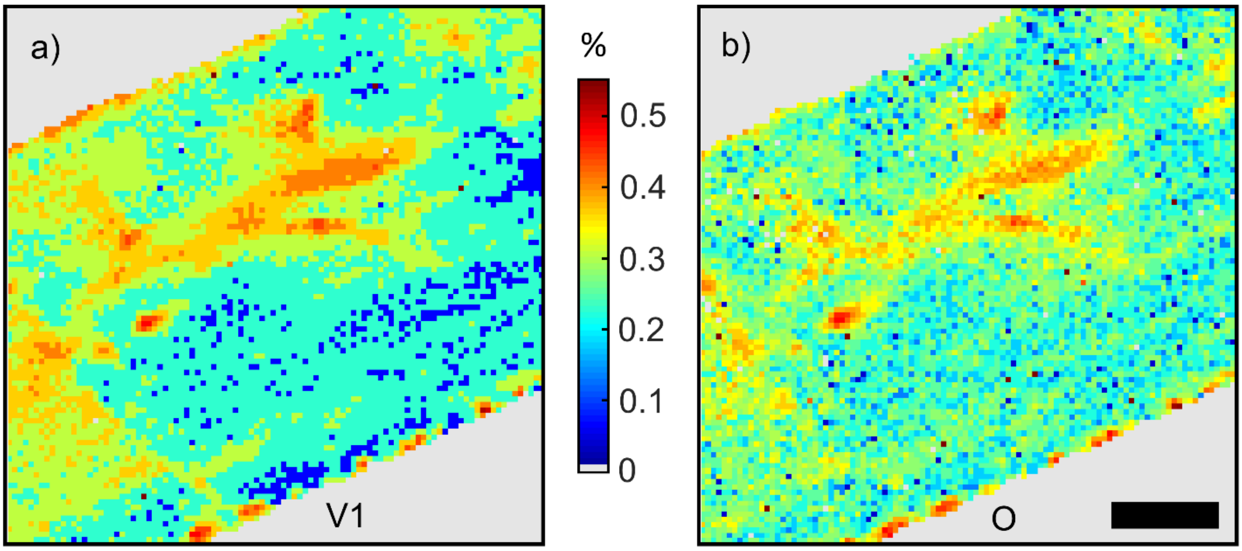

Figure S3. Relative uniaxial strain maps as estimated from the frequency shifts in the Raman modes at 193 cm^{-1} (Vanadium 1, a)) and 611 cm^{-1} (Oxygen, b)). Calibration performed using data of Ref. 3. Scale bar is 10 microns.

References

1 W. Windig, C. E. Heckler, F. A. Agblevor, R. J. Evans, R. J. *Chemometrics and Intelligent Laboratory Systems* 1992, 14, 195.
2 a) W. Windig, D.A. Stephenson, *Analytical Chemistry* 1992, 64, 2735; b) K. Artyushkova, J. E. Fulghum, *Journal of Electron Spectroscopy and Related Phenomena* 2001, 121, 33; c) M. R. J. Hachey, A. Bogomolov, K. C. Gordon, T. Rades, *Raman Technology For Today's Spectroscopists* 2004, June
3 Atkin, J. M. et al. *Physical Review B* 2012, 85, 020101
